# Introduction of Community-Based Provision of Subcutaneous Depot Medroxyprogesterone Acetate (DMPA-SC) in Benin: Programmatic Results

**DOI:** 10.9745/GHSP-D-19-00002

**Published:** 2019-06-24

**Authors:** Tishina Okegbe, Jean Affo, Florence Djihoun, Alexis Zannou, Odilon Hounyo, Gaston Ahounou, Karamatou Adegnika Bangbola, Nancy Harris

**Affiliations:** a FHI 360, Washington, DC, USA.; b JSI, Research & Training Institute, Inc., Cotonou, Benin.; c Benin Ministry of Health, Cotonou, Benin.; d JSI, Research & Training Institute, Inc., Arlington, VA, USA.

## Abstract

The Republic of Benin faces high maternal, newborn, and child mortality; low modern contraceptive use; and a critical shortage of health workers. In 2013, the Government of Benin made 3 reproductive health commitments to improve national health indicators, including expanding provision of family planning services at the community level through task sharing. Since 2016, the Advancing Partners & Communities (APC) project has been helping the Benin Ministry of Health (MOH) provide subcutaneous depot medroxyprogesterone acetate (DMPA-SC; brand name Sayana Press) through facility-based health care providers and community health workers known as *relais communautaires* (RCs). DMPA-SC is an easy-to-administer, discreet injectable contraceptive that provides 3 months of protection from pregnancy. Beginning in May 2017, the government introduced DMPA-SC through a phased approach in 10 health zones, which encompassed 149 health centers and 614 villages. Between June 2017 and June 2018, the MOH and APC trained 278 facility-based providers and 917 RCs to provide DMPA-SC, and nearly 11,000 doses were subsequently administered to 7,997 women at facilities and in communities. This article presents findings from an assessment of community-level and health facility service data collected during the first 13 months of DMPA-SC introduction in Benin. Because of this intervention, nearly 35,000 women received family planning counseling and 7,997 women chose DMPA-SC. At the community level, 3,111 DMPA-SC users were first-time users of modern contraception. The initial success of the DMPA-SC rollout in Benin shows promise for helping the country meet its reproductive health commitments.

## Abstract (precis)

Lay community health workers and facility-based health care providers in Benin were trained to administer DMPA-SC safely and effectively in 10 health zones. Community-based DMPA-SC was popular, particularly among new users of contraception, and could help the country achieve its family planning goals.

## INTRODUCTION

Historically, the Republic of Benin has had low family planning use. Desire for large families and myths and misconceptions about family planning contribute to a low modern contraceptive prevalence rate of 12% and a high total fertility rate of 5.7.^1^ Yet while conservative social norms still hinder family planning use, the modern contraceptive prevalence rate has doubled since 2006, when it was 6.1%. There has been no corresponding or significant decrease in the total fertility rate, which stood at 6.0 in 1996.^2^ The percentage of adolescents under 19 years of age who have begun bearing children has declined only slightly since 1996, from 26% to 20% in 2018.^1^^,^^2^ In 2018, nearly 1 in 3 women (32%) wanted to limit or space her next birth but was not using a family planning method.^1^ The current modern family planning method mix is dominated by implants (45.0%), followed by injectables (18.3%).^1^

Expanding access to contraception—especially in communities that have an unmet need and demand for it—is essential to reach the country's family planning goals. At the 2013 International Conference on Family Planning in Addis Ababa, the Government of Benin (GOB) made 3 commitments to improve reproductive health indicators.^3^ These commitments, which acknowledged the need to improve geographic access to family planning services for people who did not have it, were to increase the national modern contraceptive prevalence rate from 7.9%^4^ to 20% by 2018; offer free family planning services and commodities to adolescents by 2019; and introduce family planning at all levels of the health system, including injectable contraception at the community level. As part of its commitment to the Ouagadogou Partnership, the GOB pledged to enable 2.2 million additional women to use modern family planning by 2020.

With an expanding population of 11 million, nearly half of whom reside in rural areas with limited access to health care services, Benin continues to face high maternal and child mortality. This problem is compounded by the country's critical shortage of health workers. Other countries with similar shortages have introduced task-sharing approaches, as recommended by the World Health Organization (WHO),^5^ in which highly skilled health workers in facilities delegate less complex tasks to health workers who have fewer skills, thus better distributing the workload.

In 2014, the Benin Ministry of Health's (MOH's) Maternal and Child Health Directorate approved a pilot project to allow *aides-soignantes* to administer the 2-month intramuscular injectable Noristerat in Adja-Ouèrè commune in southeastern Benin. Aides-soignantes are a cadre of paraprofessional nurses' aides who are stationed at health centers and provide immunization and assist in health promotion campaigns in communities. Previously, contraceptives were available exclusively at health facilities, which limited rural women's access to family planning. During the 9-month pilot, aides-soignantes counseled 1,809 women on a range of voluntary family planning methods, and 449 women adopted Noristerat. The aides-soignantes referred 249 women to health centers for other methods including pills, intrauterine devices, and implants.^6^ Most clients had a positive response to the community-based delivery of family planning services. An assessment of the pilot concluded that community health workers (CHWs) can administer injectable contraceptives safely and effectively in Benin, and this evidence was the foundation for scaling up the approach.

## PROJECT DESCRIPTION

Since 2012, the Advancing Partners & Communities (APC) project, funded by the United States Agency for International Development (USAID), has worked in more than 40 countries to advance and support programs that improve the overall health of communities and other health-related indicators, especially related to family planning. In Benin, between 2015 and 2018, the APC project built the technical and organizational capacity of 3 local NGOs—Dedras, Bupdos, and Sia N'son—to train *relais communautaires* (RCs), the lowest-level CHW cadre, to provide high-quality community-level primary health services including family planning in 10 USAID priority health zones (Box 1).

BOX 1 **Relais Communautaires in Benin**

*Relais communautaires* (RCs) constitute a key component of the national community health strategy. They typically receive supervision from staff at public health facilities and training and small monthly stipends from local NGOs. RCs are volunteers chosen by residents of their villages to provide a package of high-impact health services (*paquet d'interventions à haut impact*) related to water, sanitation, and hygiene; maternal and child health; nutrition; malaria; and family planning (condoms and resupply of oral contraceptive pills). RCs are required to be proficient in French. An estimated 15,000 are deployed nationally and 2,080 work in the United States Agency for International Development's 10 priority health zones. The Ministry of Health offers RC refresher trainings every 2–3 years.

Benin has 2 types of RCs: those who provide curative services and those who provide preventive services only. Preventive RCs conduct health education sessions in their communities, while curative RCs also provide basic health care interventions and refer community members who need complex care to a local health facility. According to the national community health strategy, 1 curative RC should serve between 30 and 50 households that are 5 kilometers or more from the nearest public health facility. In practice, however, there are not enough trained RCs to fulfill this ideal.

The APC project built the capacity of local NGOs to train community health workers to provide high-quality community-level primary health services in Benin.

In 2016, the MOH authorized the introduction of DMPA-SC in health facilities and via RCs. Midwives administered DMPA-SC at health facilities, adding to their routine family planning service provision. RCs selected for training were linked to NGOs that received APC technical assistance.

DMPA-SC has been described as a “game-changing” injectable contraceptive because it is discreet and requires minimal training to administer.^7^ It is packaged as a prefilled, 3-month, all-in-one device and has the potential to greatly expand contraceptive access to women in need, particularly those in underserved areas. Recent studies have shown that CHWs can safely and acceptably administer DMPA-SC in communities.^8^^–^^10^ Studies have also shown that women can be trained to self-inject, where the practice is permitted and the product is registered as a self-injectable, which allows greater contraceptive control over their fertility.^11^^,^^12^

After authorization for DMPA-SC introduction was granted, Beninese stakeholders, including the MOH and implementing partner staff, visited DMPA-SC pilot sites in Burkina Faso and Uganda to learn from their introduction processes (Box 2). These visits illustrated the benefits of task sharing for family planning and laid the groundwork for further investments in community-based family planning in Benin. Based on what the Beninese stakeholders learned, it was decided that DMPA-SC introduction would start in the 6 health zones in which intramuscular depot medroxyprogesterone acetate (DMPA-IM) was available (i.e., where clients and health care providers were already familiar with DMPA). Further, targeted communication would be developed in those areas; specific monitoring and evaluation tools for DMPA-SC would be developed; and a pharmacovigilance strategy would be established. Finally, before the pilot started, national and local political and administrative authorities would be engaged.

BOX 2 **Guiding Lessons From PATH's 4-Country DMPA-SC Introduction**

1. Introduce DMPA-SC in health zones where DMPA-IM is already being offered—because providers and clients are already familiar with DMPA—including eligibility criteria, reinjection timeline, and side effect profiles.
2. Develop targeted, regional communication materials in accordance with local requirements and manufacturer guidelines.
3. Develop specific monitoring and evaluation tools for DMPA-SC.
4. Establish an appropriate pharmacovigilance strategy.
5. Engage national and local political and administrative authorities early on to garner buy-in.

Abbreviations: DMPA-IM, intramuscular depot medroxyprogesterone acetate; DMPA-SC, subcutaneous depot medroxyprogesterone acetate.

A national technical steering committee provided overall guidance and technical leadership for the introduction and implementation of DMPA-SC. The committee comprised technical and financial partners including APC, the United Nations Population Fund, USAID, the Benin Association for Social Marketing and Communication for Health (ABMS), the Beninese Association for Family Planning, and University Research Company's Advancing Newborn, Child, and Reproductive Health project. The committee adapted the training curriculum, job aids, and monitoring and evaluation tools, all in French, that PATH developed for DMPA-SC introduction in Senegal.

The 3-day RC training curriculum included a review of all available family planning methods (types, uses, side effects, and eligibility for and advantages of use); how to conduct family planning counseling and referrals; and how to complete data collection tools. The process of adapting and formatting the tools for Benin took 9 months and several committee meetings to solicit input and ensure that content adhered to national health policies, strategies, and norms. For example, the committee decided that DMPA-SC would be injected only in the back of a client's upper arm, rather than in the thigh or abdomen because of concerns for client privacy with male providers. (Pfizer's Sayana Press product is labeled for administration in either the thigh or abdomen; however, the product has been shown to be equally effective when injected in the back of the upper arm.^13^ The WHO endorses injecting DMPA-SC in the upper arm and acknowledges that this injection site may be more comfortable for some women.) Once adapted, a field test with selected RCs and health care providers in Savalou-Banté health zone was conducted to determine the usability of the materials. Participant feedback was incorporated into the final versions.

**FIGURE 1:**
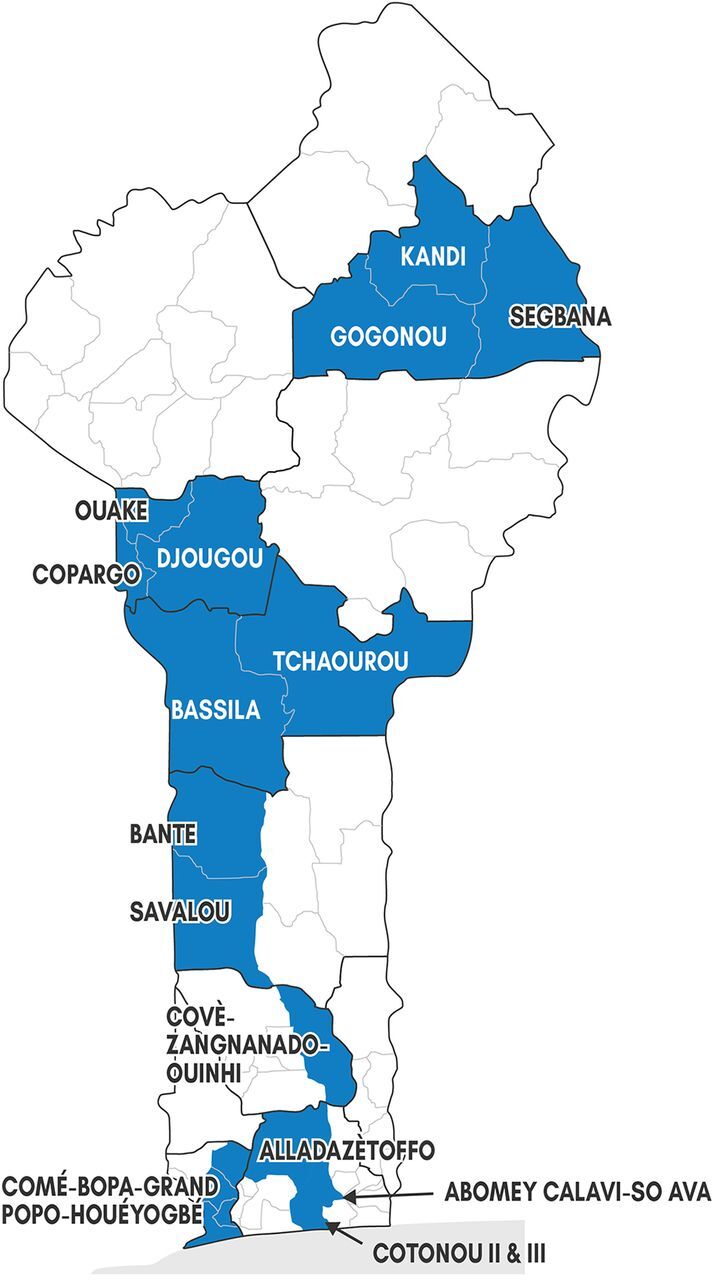
Map of DMPA-SC Introduction Zones Abbreviation: DMPA-SC, subcutaneous depot medroxyprogesterone acetate.

DMPA-SC was brought into Benin under a waiver granted by the National Drug Authority in early 2017 and was registered in July 2017. The GOB, guided by the committee, introduced DMPA-SC through public-sector health facilities, and RCs linked to those facilities used the existing infrastructure instead of an NGO-led parallel supply chain. The process required integrating DMPA-SC into national health system tools, reporting forms, and the District Health Information System 2 (DHIS 2). Beginning in May 2017, introduction proceeded in a phased rollout in 10 USAID priority health zones where RCs had been trained on family planning methods and counseling through the MOH's *paquet d'interventions à haut impact* (PIHI) curriculum (Figure 1). DMPA-SC introduction began in Abomey-Calavi health zone, followed by Djougou-Copargo-Ouaké, Kandi, Bassila, Allada Zè Toffo, Tchaourou, Comé-Bopa-Grand Popo-Houéyogbé, Cotonou II and III, Savalou-Banté, and Covè-Zangnanado-Ouinhi.

DMPA-SC introduction began in May 2017 in USAID priority health zones with RCs trained on family planning methods and counseling.

**FIGURE 2:**
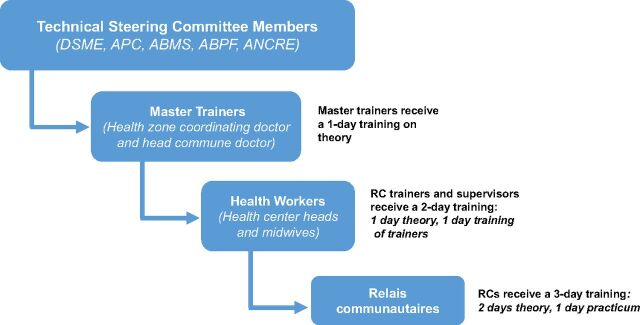
DMPA-SC Cascade Training Process Abbreviations: ABMS, Benin Association for Social Marketing and Communication for Health; ABPF, Association Béninoise pour la Promotion de la Famille; ANCRE, Advancing Newborn, Child, and Reproductive Health; APC, Advancing Partners & Communities; DMPA-SC, subcutaneous depot medroxyprogesterone acetate; DSME, Maternal and Child Health Directorate; RCs, *relais communautaires* (lay community health workers).

Health worker training was cascaded (Figure 2) to foster local ownership; increase sustainability, efficiency, and rapport between facility- and community-level health workers; and decrease costs.^14^ Technical steering committee members conducted trainings for MOH staff, who served as national master trainers. Master trainers then trained health zone staff (health center heads and midwives), who then trained RCs. Each training class of 20 RCs had 2 days of theory and a 1-day practicum at a local health center. RCs learned about all available family planning methods, including DMPA-SC, and how to refer clients who had side effects or wanted longer-acting methods. Participants practiced counseling sessions in French and the local language and gave injections to salt-filled condoms. As part of certification requirements, within 4 weeks of the training RCs were required to administer 5 supervised injections to clients who chose DMPA-SC.

After certification to provide community-based DMPA-SC, RCs received several forms of support. PIHI NGOs and RC supervisors at local health facilities held monthly group supervision sessions at which RCs discussed challenges, successes, and lessons with their peers and were reminded to follow up with clients for reinjection after 3 months. Health center in-charges also conducted quarterly on-site supervision visits with RCs. Lastly, supervision teams of staff from APC, the MOH, ABMS, and the district health zone and health center in-charges visited RCs on site 4 to 6 weeks post training to provide support and resolve challenges.

DMPA-SC is delivered to health centers through the national supply chain, which operates a “pull” system, whereby each level orders commodities; products flow from the central medical stores to district warehouses to health centers. Upon certification, health centers give RCs 5 free doses of DMPA-SC. RCs obtain product resupply at health centers at the nationally set cost of 200 CFA (∼US$0.35) per dose and resell it to clients for the same price. RCs are provided with safety boxes to dispose used DMPA-SC devices, which are discarded per medical waste disposal guidelines at the health center during routine monthly group supervision sessions.

ABMS and APC led community sensitization and national advocacy sessions to raise awareness of family planning, specifically DMPA-SC. Sensitization sessions in each introduction health zone included local elected officials and traditional and religious leaders, and featured short films on the demographic dividend, maternal mortality, and community-based injectables programming, followed by discussions. These workshops prepared traditional and political leaders to support RC DMPA-SC administration. A total of 536 local officials and 578 traditional and religious leaders participated in family planning sensitization and advocacy sessions. At the national level, ABMS and APC led briefings to inform parliamentarians and journalists of the benefits of family planning and the GOB's commitment to make DMPA-SC widely available through RCs.

The APC project assessed community-level and health facility service data collected by RCs and midwives between June 2017 and June 2018, including number of DMPA-SC doses administered, DMPA-SC uptake, and number of women receiving family planning counseling. Results and lessons from the first 13 months of DMPA-SC introduction are described below.

## RESULTS

DMPA-SC was introduced in 10 health zones in Benin through a phased approach that allowed course corrections during rollout to 149 health centers and 614 villages. The 278 trainers (health center heads and midwives) and 917 RCs who were trained administered nearly 11,000 doses of DMPA-SC over the 13-month period. RCs administered 1,309 doses of DMPA-SC to women as reinjections, and facility staff administered 1,403 doses, for a total of 2,712 reinjections. Few clients reported side effects.

The trainers and lay community health workers administered nearly 11,000 doses of DMPA-SC over 13 months.

Between June 2017 and June 2018, 9,296 women at the health facility level and 24,947 women at the community level received family planning counseling. A total of 7,997 women—including those who were adopting a modern family planning method for the first time and those switching from other family planning methods—chose DMPA-SC. DMPA-SC uptake at the community level climbed steadily during the first year of introduction and peaked in June 2018. At the facility level, DMPA-SC uptake increased from September 2017 onward, peaking at nearly 600 new users in June 2018.

**FIGURE 3:**
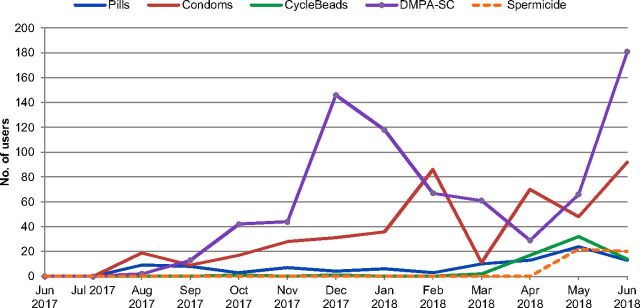
Number of Family Planning Users Switching to a New Method at the Community Level, by New Method Abbreviation: DMPA-SC, subcutaneous depot medroxyprogesterone acetate.

**FIGURE 4:**
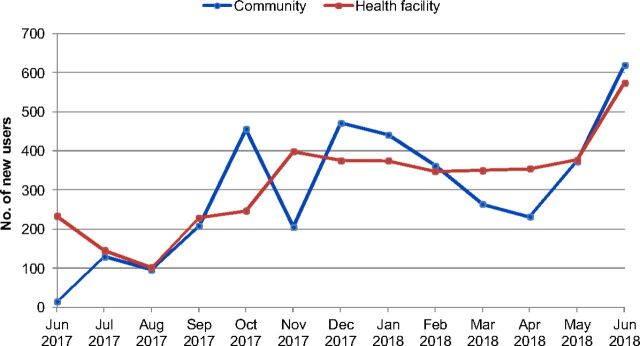
Number of New Users of DMPA-SC, by Location Abbreviation: DMPA-SC, subcutaneous depot-medroxyprogesterone acetate.

At the community level, 3,111 women were first-time users of modern contraception, and 769 women switched to DMPA-SC from another contraceptive method (Figure 3). Further, Figure 3 shows that of the 5 family planning methods available at the community level through RCs (DMPA-SC, pills, CycleBeads, condoms, and spermicide), DMPA-SC was the most popular method for current users of family planning to switch to, representing nearly 54% of all switchers. It was not possible to disaggregate how many women adopting DMPA-SC at the facility level were new users of family planning versus switchers. Figure 4 shows that for much of the year, overall DMPA-SC uptake at the community level was comparable with facility-level uptake.

**FIGURE 5:**
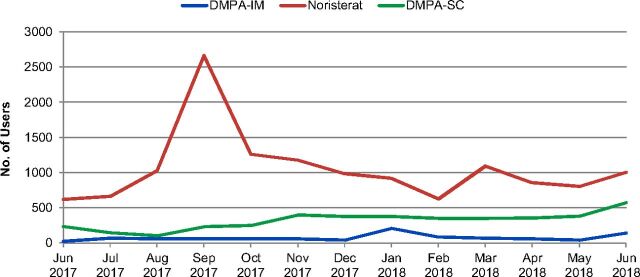
Number of Users of Injectables at the Facility Level, by Method Abbreviations: DMPA-IM, DMPA-SC, intramuscular depot medroxyprogesterone acetate; DMPA-SC, subcutaneous depot medroxyprogesterone acetate.

During the same time frame, Noristerat uptake at the health facility level was high, with 13,698 women adopting the method, followed by DMPA-SC (4,117 women) and DMPA-IM (972 women) (Figure 5). Noristerat is the most popular injectable contraceptive choice in Benin, so it is unsurprising that there were fewer users of DMPA-IM and DMPA-SC during this period. However, the goal of DMPA-SC introduction is not to supplant current options; it is to expand client access and method choice.

Although stock-outs were not measured systematically, anecdotal reports suggest they occurred at both the facility and community levels during the first months of DMPA-SC introduction. In facilities, stock-outs likely occurred because staff had to adapt to their new and additional responsibility for reordering DMPA-SC from district warehouses. In communities, either RCs did not realize that they should seek DMPA-SC resupply from the health center or when they did seek it, health centers were out of the product. It is important to note that RCs must contend with frequent stock-outs of products related to other public sector health interventions because Benin still struggles to maintain its national health commodity supply chain.

## DISCUSSION

DMPA-SC was introduced through the public sector at health centers and in communities to expand family planning access to and method choice for women. RCs administered 49% of the total DMPA-SC doses to women who chose the method. Furthermore, 80% of DMPA-SC doses at the community level were administered to first-time family planning users, suggesting that community-based distribution of DMPA-SC by RCs is an effective way to increase use of family planning. Although there were challenges throughout the process, the phased introduction allowed course corrections along the way, providing useful lessons for future DMPA-SC programming.

80% of DMPA-SC doses at the community level were administered to first-time family planning users.

Community-based distribution of injectables via CHWs is a promising way to expand access to family planning services and commodities, particularly for women who live in remote areas. Our results are in line with findings from DMPA-SC introduction in Burkina Faso, Niger, Senegal, and Uganda, which demonstrated that community-based distribution of DMPA-SC has the potential to reach new acceptors of family planning as well as youth and adolescents.^8^^–^^10^

The introduction of DMPA-SC corresponded with an increase in the number of women receiving family planning counseling at both the community and facility levels. As this experience shows, adding a contraceptive option to the method mix often prompts programs to provide refresher trainings for providers to strengthen skills, thus enhancing the overall quality of the family planning program. Many RCs reported being happy with their new DMPA-SC-related responsibilities because they were providing a useful service to community members and were eager to continue.

### Lessons Learned

The lessons learned during DMPA-SC expansion in Benin were used to improve subsequent trainings and strengthen implementation and can provide guidance to countries introducing DMPA-SC (Table).

**TABLE.** Challenges and Lessons Learned

What Was the Challenge?	Why Was This a Challenge?	How Was the Challenge Addressed? (Lesson Learned)
RC certification process took longer than anticipated	The process may take longer than expected for several reasons, including low client uptake for the new method, which prevents RCs from demonstrating competence. In addition, RCs may need additional support after initial training to manage their new duties (counseling and filling out data collection tools).	Build in “extra” time for RC certification because several factors can affect the process. Invite health zone coordinating doctors to attend supportive supervision visits to oversee health center staff who supervise RCs. (*Note that current global guidance states that in countries where CHWs already administer injectables, certification can be granted with 3 injections)*
Community awareness about DMPA-SC was low in regions where introduction occurred	Communication campaign was not launched simultaneously with the introduction of DMPA-SC.	Introduce a targeted communication campaign in regions where DMPA-SC is introduced to garner interest and create awareness about the method.
Stockouts of DMPA-SC at the facility and community level were frequent at the beginning of introduction	Health facility staff incorrectly assumed that the APC project was responsible for reordering DMPA-SC stock.	Clearly outline health facility manager responsibilities during training and encourage staff to take ownership for ordering commodities. At the national level, it is critical to ensure that the method appears on the national resupply order form as soon as the new method becomes available.
RC family planning counseling skills were low	RCs previously had been trained on family planning through MOH PIHI curriculum and initially APC only provided a short counseling skills session during the DMPA-SC training.	Include a half-day refresher training session on counseling skills so that all RCs have an opportunity to practice and enhance their counseling skills. A counseling job aid was also added to guide RCs through the counseling process and ensure informed choice and voluntarism.
Data collection was not standardized across RCs or health facilities	During the first few months of DMPA-SC introduction, RCs did not receive family planning registers (which were to be provided by their linked NGO) and therefore did not collect data in a systematic way.	During subsequent RC trainings, the importance of using the appropriate forms to collect client data was emphasized. Additionally, time was allotted for RCs to practice filling in the client family planning registers. At the health facility level, technical assistance is provided to providers during routine supervision visits to ensure that they are correctly collecting and reporting family planning data.

Abbreviations: APC, Advancing Partners & Communities; CHW, community health worker; DMPA-SC, subcutaneous depot medroxyprogesterone acetate; MOH, Ministry of Health; PIHI, *paquet d'interventions à haut impact* (package of high-impact health services); RC, *relais communautaires* (lay community health workers).

#### Build Facilitation Skills in CHW Trainers

Master trainers realized that many RC trainers had little experience facilitating sessions, which resulted in a low-quality training experience for RCs. The MOH and APC added a module on how to conduct effective trainings to the RC trainer curriculum. This half-day training increased RC trainer competency and led to higher-quality RC training sessions.

#### Pay Attention to Training Needs and Be Able to Provide Additional Support

During the post training supervision phase, many RCs displayed weak family planning counseling skills. To remedy this problem, the APC project developed a job aid that gave concise guidance on how to counsel on all family planning methods and ensure informed choice and voluntarism. This job aid was given to trained RCs and incorporated into the training curriculum. A half-day counseling refresher session was added to subsequent trainings to supplement the family planning information that RCs received under the government's PIHI curriculum. The refresher training included time to review the job aid and practice its contents through role play. Lastly, a day of on-site counseling capacity building for RCs who continued to exhibit poor counseling skills post training and certification was added to MOH, APC, and ABMS routine monitoring and supervision visit schedule.

#### Build in Ample Time to Certify Community Health Workers

RC certification took longer than expected, sometimes exceeding the 4-week validation period initially established. Of the 917 RCs trained, approximately half were certified to administer DMPA-SC by June 2018. Those who did not become certified within 2 months of training are preventive RCs, conducting family planning and DMPA-SC awareness-raising activities in their communities. One cause of delayed certification was supervisors' lack of adequate follow-up with RCs. In response, APC invited health zone coordinating doctors, who oversee health center staff, to attend the supportive supervision visits so they could note challenges, develop monitoring and action plans, and encourage supervisors to be available to RCs.

#### Recognize That Appropriate and Simultaneous Education and Communication Initiatives Are Critical to Family Planning Uptake

Another cause of delayed RC certification was low client uptake of DMPA-SC, which prevented RCs from administering the 5 voluntary client injections to demonstrate competence. Low client uptake of an unfamiliar (i.e., new) family planning option is common. To rectify this, RCs strengthened their mobilization efforts to challenge social norms that hinder family planning uptake. Client demand was also low, likely due to the delayed launch of a mass media DMPA-SC educational campaign. It is critical to launch corresponding communication campaigns (in accordance with local requirements and manufacturer guidelines) and product introduction simultaneously to create momentum and attract clients. The campaign should include information on the benefits and risks of family planning in general and the new method in particular, as well as where to access it.

It is critical to launch communication campaigns and product introduction simultaneously.

National health worker strikes, which took place during RC training, also delayed RC certification. Facility-level health care providers serve as RC supervisors, so when they were unavailable during the strike, RCs were unable to complete the requisite number of client injections for certification. Many trained RCs were unable to provide community-based services during this period, either because they were not yet properly certified to do so, were not being directly supervised, or were not able to resupply stock.

#### Provide Sufficient Training to Enable Facility-Based Providers to Order Commodities Early and Ensure That MOH Includes New Commodities on Resupply Forms at the Onset of Product Introduction

Product stock-outs were initially problematic in the first health zone because health center staff were unaware of their responsibility for reordering commodities. The reason for this is likely twofold: (1) health center staff assumed that because APC was leading the DMPA-SC trainings, the project was also responsible for product resupply; and (2) the national commodities resupply form was not updated to include DMPA-SC until June 2017. Stock-outs at the facility level trickled down and affected community-level provision because RCs sought DMPA-SC resupply at health centers.

A continuing challenge is the collection of high-quality data. RCs record community-level data in family planning registers, which are compiled at the facility level. During the early phases of implementation, some RCs did not have the registers because of delayed printing by the affiliated NGOs and therefore did not collect or record data in a standardized manner. Once those RCs received their registers, APC and MOH staff provided technical support during monitoring visits to ensure data were collected properly. Subsequent trainings emphasized the importance of monitoring, evaluation, and data collection and allowed time for RCs to practice completing the data registers. Data quality also remains a problem at the health facility level because reporting is primarily paper based, which often results in poor record keeping. APC and MOH staff provide technical assistance to facility-level health care providers during routine supervision visits to ensure that family planning data are captured and reported accurately. High-quality data are critical for measuring the impact of DMPA-SC introduction in the health zones.

Collection of high-quality data is a continuing challenge, and more data on side effects experienced by clients are needed.

Another ongoing challenge is limited data on client side effects. Women rarely reported side effects to RCs or facility-level health care providers, and although it is possible that this reflects a low rate of experienced side effects, it is also possible that clients fail to report side effects that they consider “minor” or health workers are not collecting or reporting this information accurately. We suggest establishing a more robust system to monitor and capture potential minor and major side effects.

Our final lesson is the importance of involving communities and their gatekeepers. We found that inviting local stakeholders—including religious and traditional leaders, local and national politicians, and communities at large—to orientation sessions during the DMPA-SC introduction process was critical to gaining their support, without which the program would not have succeeded.

### Limitations

A limitation of this analysis is the lack of unique client identifiers within the data. Consequently, it is impossible to track individual women over time to determine if they elected to be reinjected with DMPA-SC and if so how many times; therefore, calculating continuation and discontinuation rates is also impossible. The indicators simply captured the total number of reinjections in communities and at health facilities during the specified period. Moreover, this type of routine service statistics data does not measure the number of contraceptive discontinuers, only users, so it is difficult to assess if and how much the program increased contraceptive use and contributed to national reproductive health goals. Further, because this analysis is restricted to service statistics data, client motivations for using family planning for the first time or choosing to switch to DMPA-SC from another method cannot be determined.

Additionally, given the MOH's current facility-level indicators, it is impossible to determine if women adopting DMPA-SC were new users to family planning or had switched from another method. Having this disaggregated information in the future will help determine user profiles for women who choose DMPA-SC. The data show that in health centers where DMPA-SC and DMPA-IM were both offered, DMPA-SC was the more popular method, surpassing the number of DMPA-IM users during each of the 13 assessed months. It would be helpful to monitor from which methods users were switching to determine if DMPA-SC introduction decreases use of other methods, including DMPA-IM and Noristerat. Further, age-disaggregated data are not collected at either the facility or community level because the indicator has yet to be added to national data collection forms. This information is critical for improving youth-focused programming.

Having disaggregated information would help monitor family planning method preferences and improve youth-focused programming.

## CONCLUSIONS

Results from the first 13 months of DMPA-SC introduction in Benin demonstrate the demand for community-based family planning provision, as uptake of the injectable contraceptive increased steadily at the community level. Between June 2017 and June 2018, 7,997 women chose to adopt DMPA-SC as their preferred method of family planning, from either a community- or facility-based health worker. Results suggest that service provision through RCs is feasible and acceptable among clients, as nearly 4,000 women—nearly 80% of whom were first-time users of modern contraception—chose to receive this method at the community level.

As the popularity of DMPA-SC grows in Benin, service provision through additional channels, including self-injection, should be considered to make the method available to all who would chose it. Recent findings from a DMPA-SC self-injection trial in Malawi show a user discontinuation rate of 56% when CHWs administer injections. However, when women self-inject, the discontinuation rate drops by almost half, to 30%.^15^ Although DMPA-SC self-injection is not yet authorized in Benin, the GOB committed in 2018 to expanding access to DMPA-SC by scaling the method in the public sector to all 34 health zones, introducing it in the private sector, and piloting self-injection. Planning discussions are ongoing, and lessons from this introduction experience in 10 health zones will inform continued DMPA-SC expansion and scale up in Benin.

*As a midwife, I approve this initiative because it solves the problem of geographical accessibility because the health centers often serve remote villages. The* relais communautaires *were chosen on the basis of certain well-defined criteria and they have been trained to offer Sayana Press, so for me they have the competence. [Health care provider]*

*I am happy to have been chosen and trained to offer this service because now women in my community no longer need to pretend to take their children for care at the health center to receive a contraceptive method, [they] now can access a method close to home. [*Relais communautaire*]*

## Notes

**En français**

**Introduction de l'offre communautaire du Sous-cutané Depot Medroxyprogesterone Acetate (DMPA-SC) au Bénin : résultats programmatiques**

Les agents de santé communautaires et les prestataires de soins de santé des formations sanitaires publiques au Bénin ont été formés pour administrer le DMPA-SC de manière sûre et efficace dans 10 zones sanitaires. Le DMPA-SC à base communautaire est populaire, en particulier parmi les nouveaux utilisateurs de contraception, et peut aider le pays à atteindre ses objectifs en matière de planification familiale.

**RÉSUMÉ**

La République du Bénin est confrontée à une forte mortalité maternelle, néonatale et infantile; faible utilisation de contraceptifs modernes; et une pénurie critique d'agents de santé. En 2013, le gouvernement du Bénin a pris 3 engagements en matière de santé reproductive afin d'améliorer les indicateurs de santé nationaux, notamment dans l'expansion de la fourniture de services de planification familiale au niveau communautaire par le biais d'une délégation des tâches. Depuis 2016, le projet Advancing Partners & Communities (APC) aide le Ministère béninois de la Santé (MS) à fournir de l'acétate de médroxyprogestérone-dépôt sous-cutané (DMPA-SC; nom de marque Sayana Press) par l'intermédiaire des prestataires de soins de santé des formations sanitaires et d'agents de santé communautaires connu sous le nom de relais communautaires (RC). Le DMPA-SC est un contraceptif injectable discret, facile à administrer, qui offre une protection contre la grossesse pendant 3 mois. À partir de mai 2017, le gouvernement a introduit le DMPA-SC selon une approche progressive dans 10 zones sanitaires comprenant 149 centres de santé et 614 villages. Entre juin 2017 et juin 2018, le MS et APC ont formé 278 prestataires des formations sanitaires et 917 RC à fournir du DMPA-SC, et prés de 11 000 doses ont ensuite été administrées à 7 997 femmes dans les formations sanitaires et dans les communautés. Cet article présente les résultats d'une évaluation des données sur les services au niveau communautaire et des formations sanitaires collectées au cours des 13 premiers mois de l'introduction du DMPA-SC au Bénin. Grâce à cette intervention, près de 35 000 femmes ont bénéficié de conseils en matière de planification familiale et 7 997 d'entre elles ont choisi d'utiliser DMPA-SC. Au niveau communautaire, 3 111 utilisatrices du DMPA-SC utilisaient pour la première fois la contraception moderne. Le succès initial du déploiement du DMPA-SC au Bénin montre qu'il est prometteur d'aider le pays à respecter ses engagements en matière de santé de la reproduction.

**Peer Reviewed**

**First Published Online:** June 6, 2019

**Funding:** This work was made possible by the generous support of the American people through the United States Agency for International Development (USAID) and implemented in collaboration with the Beninese Ministry of Health (MOH). Financial assistance was provided through the Advancing Partners & Communities (APC) project funded by USAID under Agreement No. AID-OAA-A-12-00047, beginning October 1, 2012. APC is implemented by JSI Research & Training Institute in collaboration with FHI 360.
